# Viral load and high prevalence of HR-HPV52 and 58 types in black women from rural communities

**DOI:** 10.1186/s12879-021-06042-6

**Published:** 2021-04-17

**Authors:** Lays Paula Bondi Volpini, Jerusa Araújo Dias, Luciana Bueno de Freitas, Maria Carmen Lopes Ferreira Silva, Angélica Espinosa Miranda, Liliana Cruz Spano

**Affiliations:** 1 Infectious Diseases Post-Graduate Program, Federal University of Espírito Santo, Vitória, Brazil; 2 Department of Nursing, University Center of Northern Espírito Santo, Federal University of Espírito Santo, São Mateus, Brazil; 3 Multivix Faculty, Vitória, Brazil; 4 Department of Pathology, Center of Health Sciences, Federal University of Espírito Santo, Vitória, Brazil; 5 Department of Social Medicine, Center of Health Sciences, Federal University of Espírito Santo, Vitória, Brazil

**Keywords:** HPV, Viral load, Rural communities, Cervical cancer screening

## Abstract

**Background:**

The high-risk human papillomavirus (HR-HPV) infection is the main cause of cervical cancer development, and the most common types were included in the last approved nonavalent vaccine (9vHPV). Geographical, socioeconomic and ethnic barriers in developing countries challenge primary and secondary prevention measures of cervical cancer. We aimed to determine the prevalence of HPV infection and the viral load of HR-HPV 9vHPV-related types black women resident in rural semi-isolated communities.

**Methods:**

A descriptive study was conducted with 273 cervical samples of women from rural communities of Southeastern Brazil. Viral DNA was amplified by PCR, the genotype was identified by Reverse Line Blot (RLB) and Restriction Fragment Length Polymorphism (RFLP), and real-time PCR was applied to determine the viral load.

**Results:**

HPV frequency was 11.4% (31/273), associated with the presence of cytological abnormalities (32.3%; *p* < 0.001). Thirty-one distinct genotypes were detected; HR-HPV occurred in 64.5% (20/31) of the samples and the most prevalent type were HPV52 > 58, 59. Multiple infections occurred with up to nine different genotypes. The viral load of HR-HPV 9vHPV-related types was higher in lesions than in normal cytology cases (*p* = 0.04); “high” and “very high” viral load occurred in HSIL and LSIL, respectively (*p =* 0.04).

**Conclusions:**

We highlight that despite the low HPV frequency in the black rural women population, the frequency of HR-HPV was high, particularly by the HR-HPV52 and 58 types. Moreover, the HR-HPV viral load increased according to the progression from normal to lesion, being a potential biomarker to identify those women at higher risk of developing cervical lesions in this population.

## Background

Human papillomavirus (HPV) is the most common sexually transmitted infection (STI), and the main cause of cervical cancer development [1]. According to the risk of oncogenic progression, HPV with tropism for the anogenital site are classified as low-risk (LR-HPV), high-risk (HR-HPV), probable carcinogenic and undetermined-risk (UR-HPV) [2]. Among HR-HPV strains, the HR-HPV16 and 18 are responsible for the most cases of cervical cancer, followed by types 31, 33, 45, 52 and 58 [3].

Cervical cancer is the third most common cancer among women worldwide, with an estimated 569,847 new cases, and 311,365 deaths in 2018 [4]. More than 84% of cases and deaths due to cervical cancer occur in women from low-resource regions of the World [5]. Control measures are based on the primary prevention with the bivalent HPV (16/18), tetravalent HPV (4vHPV- 6/11/16/18), and nonavalent (9vHPV- 6/11/16/18/31/33/45/52/58) vaccines, and on the secondary prevention by Pap test screening for precursor lesions, which reduced the cervical cancer in developed countries with organized screening programs [6]. Additional secondary tests based on the detection of HPV DNA have been introduced in the cervical cancer screening associated with cytology (co-test) or as primary screening in some developed countries [7]. Although Brazil has adopted Pap test as control measure for decades, besides 4vHPV since 2014, cervical cancer remains the third most common cancer in women, with approximately 16,370 new cases in 2018, corresponding to 41% of all cervical cancers cases in South America [4, 8].

Geographic, socioeconomic and ethnic barriers contribute to inequalities in the access to healthcare, as observed in black women, with a low socioeconomic status [9]. In this context are the Brazilian black women who live in rural African slave remnant communities, which health data are limited, especially regarding HPV prevalence and risk factors associated with cervical cancer [10, 11].

Additional strategies for cervical cancer prevention are imperative. Despite the low specificity, the HPV test has high sensitivity, allowing larger screening intervals and playing a promising role in cervical cancer prevention [12]. The association with potential biomarkers of high-grade cervical lesion (HSIL) and cancer, such as HPV viral load, could improves the specificity and will have potential benefit in reducing the incidence of cancer [13].

In this study, we aimed to determine the prevalence of HPV infection and the viral load of the HR-HPV genotypes correspondent to the types of the 9vHPV (9vHPV-related) in black women resident in rural semi-isolated communities.

## Methods

### Patients, samples, sociodemographic data and ethic aspect

This is a descriptive study conducted between March 2016 to August 2017 in sexually active women from 16 semi-isolated communities located at Espírito Santo State, Southeastern Brazil. These communities are connected by unpaved roads of difficult access, between 6.21 mi (10 km) and 18.64 mi (30 km) from urban centers and from each other.

Women from 15 to 79 years, sexually actives, were included in this study. They answered a questionnaire about sociodemographic and behavioral data. Cervical samples were collected using a cytobrush, transported in Digene Specimen Transport Medium (STM, Qiagen Incorporated, Valencia, CA), and maintained at − 70 °C for HPV investigation. Papanicolaou method was applied for the cytological evaluation at the Pathology Laboratory of the University Hospital of the Espírito Santo state by a single pathologist with expertise, and cervical abnormalities were interpreted according to the criteria defined in the Bethesda system [14].

This research obtained approval by the Ethical Research Council of the Center of Health Sciences of the Federal University of Espírito Santo, Brazil (Protocols n. 1.308.539 and 2.925.384). All participants signed an informed consent agreement. All the minors enrolled in this study and their parents/guardians signed the agreement consent (Resolution 466/12 of the National Health Council and its complementary) after the explanation of the study objectives.

### HPV detection and genotyping

DNA was obtained using the QIAamp DNA Mini Kit (Qiagen, Valencia, CA, USA) according to the manufacturer’s instructions, and the HPV DNA was detected by amplification with the PGMY09/11 set of primers [15]. PCR for the *β-globin* gene was performed in HPV negative samples as an extraction control and to assess the DNA integrity [16]. All the HPV positive samples were genotyped using a Reverse Line Blot (RLB) assay and Restriction Fragment Length Polymorphism (RFLP), as previously described [17, 18].

HPV genotypes identified in this study were classified as HR-HPV (16, 18, 31, 33, 35, 39, 45, 51, 52, 56, 58, 59), LR-HPV (6, 11, 40, 42, 43, 44, 54, 61, 70, 72, 81 and 89) and UR-HPV (2a, 3, 7, 10, 27, 28, 29, 30, 32, 34, 55, 57, 62, 67, 69, 71, 74, 77, 83, 84, 85, 86, 87, 90 and 91) [2]. Genotypes considered probable carcinogenic (26, 53, 66, 68, 73 and 82) were grouped with HR-HPV.

### Quantitative real-time PCR analysis for HPV DNA viral loads

The viral load of HR-HPV 9vHPV-related types (16, 18, 31, 33, 45, 52 and 58) was determined by quantitative real-time PCR (qPCR). Primers and probes for *E6* and *E7* genes were used for HPV16 and HPV18, respectively, and *E7* for HPV31, 33, 45, 52 and 58 [19, 20].

The assay for HPV16 and 18 was performed according to Gravitt et al. (2003) [19] with modifications. In brief, 2 μl of extracted DNA were added to 8 μL of master mix containing 1X Buffer (200 mM Tris HCl and 500 mM KCl), 20 mM of each dATP, dGTP, dCTP and dTTP, 0.1 μM of hydrolysis probe, 0.2 μM of each primer, 0.6 mM of MgCl_2_, 0.12 μM of CXR Reference Dye (Promega Madison, WI, USA), and 1 Unit of Platinum Taq DNA Polymerase (Invitrogen Carlsbad, CA, USA). Thermal cycler conditions were previously described [19]. The assay for HPV 31, 33, 45, 52, and 58 was performed using 1x TaqMan® PCR Master Mix (Thermo Fisher Scientific, Waltham, MA, USA), as previously described [20].

The viral load was normalized using qPCR for the *β-globin* gene with primers described by Huang et al. (1989) [16]. The hydrolysis probe was designed to target the region between the primers using Primer3 v.0.4.0 [21]. The reaction condition proceeded as above described. All amplification assays were carried out on StepOnePlus equipment (Applied Biosystems, Foster City, CA, USA). Standard curves for absolute quantification of HPV types and *β-globin* were generated in 10-fold serial dilutions (10^6^–10^1^ copies of genome) with synthetic oligonucleotides fragments of 150 bp (Thermo Fisher), designed from reference sequences of HPV genotypes and *β-globin* spanning the region between primers (GenBank ID: HPV16 - K02718, HPV18 - X05015, HPV31 - J04353, HPV33 - M12732, HPV45 - X74479, HPV 52 - X74481, HPV58 - D90400, *β-globin -* NC_000011.10). The viral load, expressed in copies per cell (c/c) was obtained dividing HPV copy number by half of the *β-globin* copy number, and was categorized as low (1–10 c/c), moderate (11–100 c/c), high (101–1000 c/c) or very high (> 1000 c/c) [22]. HPV viral load was log-transformed (log_10_) to graphic representation.

### Statistical analysis

Statistical analyses were performed using SPSS 20.0 software for Windows (SPSS, Inc., Chicago, IL). Groups were compared using the Chi-square test or the Fisher exact test for qualitative characteristics, and using the Mann–Whitney and Kruskal Wallis tests for continuous ones. A *p*-value of less than 0.05 was considered statistically significant.

## Results

A total of 273 black women participated in this study and HPV DNA was detected in 11.4% (31/273) of them. The behavioral and demographic information about the women enrolled in the study are shown in Table 1. The age of patients ranged from 15 to 79 years, with a mean age of 41.9 ± 14.2 years.

**Table 1:** Behavioral and demographic characteristics of black women from rural communities of Espírito Santo, Brazil (*n* = 273)

Characteristics	HPV negative (%) ***n*** = 242	HPV positive (%) ***n*** = 31	p
**Distance from urban center (miles)**			
≤ 6.21	45 (88.2)	6 (11.8)	0.92
> 6.21	197 (88.7)	25 (11.3)	
**Use of contraceptive (Condom)**			
Yes	65 (89)	8 (11)	0.90
No	177 (88.5)	23 (11.5)	
**Number of sexual partner (last year)**			
1	175 (87.1)	26 (12.9)	0.17
≥ 2	67 (93.1)	5 (6.9)	
**Last Papanicolaou test**			
≤ 12 months	50 (96.2)	2 (3.8)	0.09
> 12 months	192 (86.9)	29 (13.1)	

Cytological results were obtained from 263 and 7.6% (20/263) presented cytological abnormalities. Ten of HPV positive samples (32.3%) presented cytological abnormalities (OR = 10.57, 95% CI 3.95 to 28.28, *p* < 0.001).

**Fig. 1:**
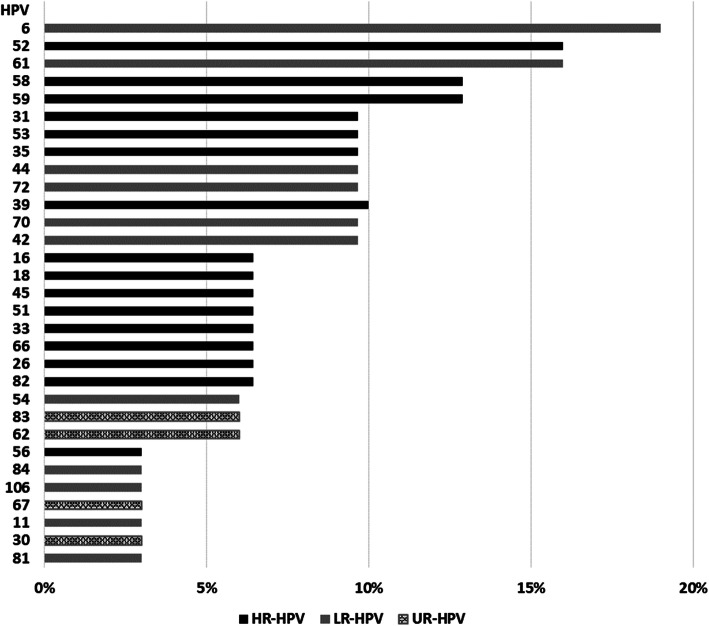
Frequency of HPV genotypes. Frequency of HPV genotypes observed in black women from rural communities of Espírito Santo, Brazil. X-axis: frequency of positive cases for each HPV genotype; Y-axis: HPV genotypes (classified by risk)

Thirty-one distinct HPV genotypes were identified, and HR-HPV occurred in 64.5% (20/31) of the samples (Table 2). HPV52 was the most prevalent HR type (16.1%), followed by 58, 59 (12.5% each), 31, 35, 39 and 53 types (9.4% each) (Fig. 1). Multiple infections occurred in 48.4% (15/31), with up to nine distinct genotypes. Considering only the HR-HPV infection, multiple types occurred in 38.7% (12/31) and, of these, 33.3% (4/12) were present in cases with cytological abnormalities. Eighty percent of multiple infections occurred in women which reported to have only one sexual partner.

**Table 2:** HPV genotypes observed in black women, according to cytological data, from rural communities of Espírito Santo, Brazil

Cytology^**a**^	No of samples	HPV genotypes^**b**^
ASCUS	3	**52** (2)/ **33**, 42, **52**, 53, **58**
LSIL	4	6/ 61/ **31**, 35, 44, 54, 70/ 6, **16**, **31**, 35, 39, 56, 61, 66, 84
HSIL	3	**16**/ **52**/ 11, 26, 35, **58**
Normal	21	6/ 53/ 54/ **58** (2)/ 61 (2)/ 62/ 72/ 83/ 6, 42/, **33**, 51/ 51, 59/ 53, 82/ 72, 81, 106/ 6, 59, 82/ **18**, **52**, 59/ 6, 30, 39, 44, 67/ **18**, 26, **45**, 59, 61/ **31**, 42, 44, 70, 83/ 39, **45**, 66, 70, 72

^a^ASCUS, atypical squamous cells of undetermined significance; LSIL, low-grade squamous intraepithelial lesion; HSIL, high-grade squamous intraepithelial lesion

^b^Number of samples in parentheses, when greater than one; Types in multiple infection are between comma; Samples are slash-separated; 9vHPV-related types are highlighted

**Fig. 2:**
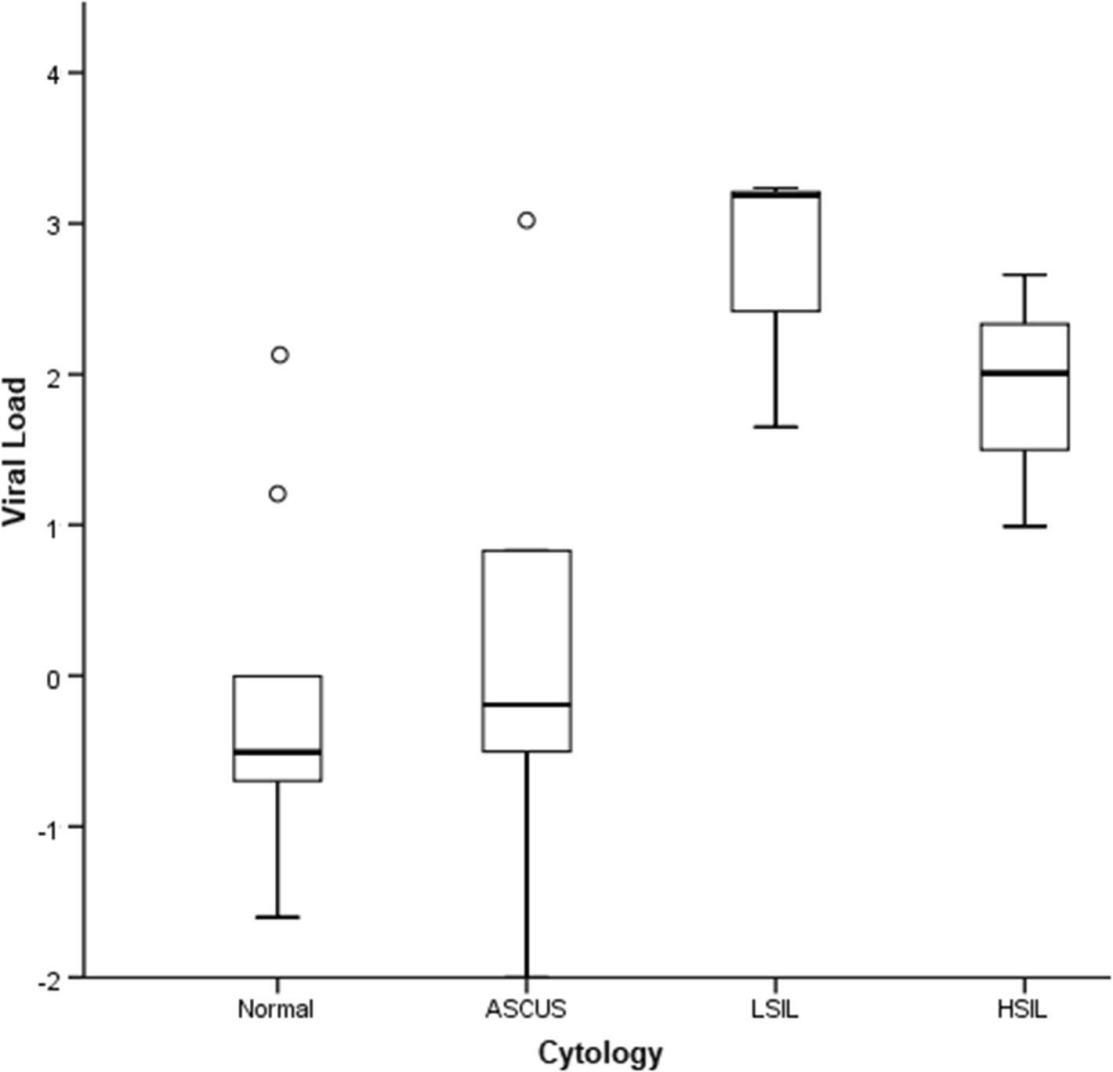
Boxplot of the HPV viral load of black rural women, according to cytological results. Distribution of HPV cases among normal, ASCUS, LSIL and HSIL cytology (*p* = 0.04). Bands, boxes, and whiskers represent the median, interquartile range and minimum and maximum values of viral loads, respectively; circles represent outliers

The viral load of HR-HPV positive samples, corresponding to the 9vHPV-related types, according to cytological results can be observed in Table 3. High and very high viral load was observed in all cytological results presenting HPV16, 31 and 52. Irrespective of genotype, high (102.0 c/c) and very high viral load (1527.54 c/c) were detected in HSIL and LSIL results, respectively, while low viral load was detected in normal (0.31 c/c) and ASCUS (0.64 c/c) results (*p =* 0.04) (Fig. 2).

**Table 3:** Viral load classification according to HR-HPV types and correspondent cytological results of black women from rural communities of Espírito Santo, Brazil

Cytology^**b**^	Viral load^**a**^	HPV type
ASCUS	Low	33, 52 (2), 58
	Very high	52
LSIL	Moderate	31
	Very high	16, 31
HSIL	Low	58
	High	16, 52
Normal	Low	18 (2), 33, 45, 58 (2), 45
	Moderate	31
	High	52

^**a**^low (1–10 c/c), moderate (11–100 c/c), high (101–1000 c/c) or very high (> 1000 c/c)

^b^ASCUS, atypical squamous cells of undetermined significance; LSIL, low-grade squamous intraepithelial lesion; HSIL, high-grade squamous intraepithelial lesion

## Discussion

Especially for disadvantaged populations, as black women living in rural areas, little is known about cervical cancer, even less about HPV infection rates and types [10, 11]. In Brazil, there are 3386 communities of African descendants of enslaved blacks, distributed in 24 states [23], and little attention is given to the issue of sexually transmitted infection [10, 11]. In this study, we first show a low frequency of HPV infection in such women population but with a diversity of types and multiple infections, and a higher frequency of the HR-HPV 52 and 58 types over the HPV 16 type. Second, we demonstrate that the viral load of HR-HPV 9vHPV-related types increase between normal and cytological lesion.

We found an overall HPV frequency of 11.4% similar to the low HPV prevalence in similar Brazilian populations, of 12.6 and 13% [10, 11]. In women living also in rural areas worldwide, regardless of ethnicity, low rate ranging 10–26% was described [24–26]. Conversely, these rates contrasts with those found in urban centers worldwide, which varied from 27.7–52,7% [27–29], in part suggesting that limited contact with urban centers could restrict the rate of HPV infection.

On the other hand, a high diversity of genotypes (*n* = 31) and multiple HPV-types infection (48.4%) occurred among the black rural women. Considering only the HR-HPV infection, multiple types (38.7%) were higher than that described in those studies with similar black rural women [10, 11]. Worldwide, multiple infection rates in women from urban centers, independent of lesion grade, have ranged from 16.7–41.8% [27–31]. There is no consensus whether the HPV types in multiple infections occur in a competitive or cooperative relationship. While Trottier et al. (2006) [32] suggest that an interaction between HPV types can increase the risk of lesion, other studies did not show an association with multiple HPV infection [33, 34].

In our study, HPV52 and 58 were the most frequent HR-HPV types, similar to those observed in black rural women of Northeastern Brazil [10, 11]. Sammarco and colleagues (2016) also found HPV58 as most frequent genotype, although in a population different from our study [35]. Interestingly, studies regarding HPV genotypes among different ethnic populations, have reported that HPV16 and 18 were less prevalent among Hispanic and non-Hispanic black women compared to non-Hispanic white women [36, 37]. These data are also supported by studies with African women from Kenya and Mozambique, which showed the HPV58 followed by HPV16, HPV53 and HPV18 types, and the HPV52 followed by HPV35, 16 and 53 as the most frequent in that population, respectively [38, 39]. However, it is unclear why these women are less likely to be infected with HPV16 and 18. Vidal et al. (2014) [36] suggest that African descendant women, which harbor others HR-HPV than HR-HPV16 and 18, maybe more resistant or have lower exposure to infection by HR-HPV16 and 18. Other factors, as the composition of vaginal and cervical microbiota, may influence on the acquisition of certain types of HR-HPV, playing a role in the persistence of HPV and development and progression to cervical lesion [40, 41]. Besides, the ethnicity/race may influence the vaginal microbiota and *Lactobacillus*-dominated cervical microbiota is less common among African women [42–44].

Our results also draw attention to a greater benefit in the use of the 9vHPV vaccine to the detriment of the 2vHPV or 4vHPVin the prevention of cervical cancer in black women, at least from rural communities, because it contemplates the most frequent HR-HPV types that infect the black rural population (HPV52 and 58). Therefore, these findings may contribute with public health politics regarding the election of vaccines for specific populations.

We also showed that the viral load of HR-HPV 9vHPV-related types was higher in cases with cervical lesion than in normal cytology (*p* = 0.04). Most of the studies investigated HPV16 viral load, and some of them showed the association between an increase of the viral load and the cervical lesion [45, 46]. The data on the viral load of HR-HPV other than HPV16 also showed similar results [13, 47, 48]. It is noteworthy that different HPV genotypes may have different mechanism to develop a high-grade cervical lesion and the viral load can modify the risk of precancerous disease [33]. This fact was demonstrated by Adcock and colleagues that, considering only the genotypes, the HPV16, 33 and 31 had greatest risk of pre-cancerous disease, and high viral loads for HPV18, 35, 52 and 58 carried more risk than intermediate levels for HPV16, 31 and 33 [33]. It is important to highlight that we observed a high and very high viral load of HPV 52 (the most frequent HPV genotype in this population) in normal and ASCUS results in women with a mean age of 42 years, living in semi-isolated regions and with little access to health services. According to Brazilian guidelines for cervical cancer screening, repeat cytology is the management for such cytological results. However, considering our population studied and the low sensitivity of cytology, the HR-HPV test carried out simultaneously with viral load test could be useful to identify these women at higher risk of developing cervical lesions, increasing the interval of screening or referring them to immediate colposcopy instead repeat cytology.

This study had some limitations as the lack of cytological data for all participants, the absence of cervical histologic data, to confirm the precancerous lesions, as well as the small sample size due to difficulty of access to rural communities. However, we believe that the data provide valuable information about the profile of HPV infection in such population.

## Conclusions

In conclusion, our data show a low frequency of HPV infection in black women from rural semi-isolated communities, and the HR-HPV52 and 58 as the most types in contrast to HPV16. We also demonstrate an increase of HPV viral load of the 9vHPV-related types with cervical lesions cases. We believe that these findings may assist in the strategies for cervical cancer prevention, especially for disadvantaged populations.

## Data Availability

The data sets used and analyzed during the current study are available from the corresponding author on reasonable request.

## Abbreviations

9vHPV: Nonavalent HPV vaccine
ASCUS: Atypical squamous cells of undetermined significance
c/c: Copies per cell
HSIL: High-grade squamous intraepithelial lesion
HPV: Human papillomavirus
HR-HPV: High-risk HPV
LR-HPV: Low-risk HP
qPCR: Quantitative real-time PCR
RFLP: Restriction Fragment Length Polymorphism
RLB: Reverse Line Blot
STI: Sexually transmitted infection
UR-HPV: Undetermined-risk HPV
